# Spatial Association of Urban Form and Particulate Matter

**DOI:** 10.3390/ijerph18189428

**Published:** 2021-09-07

**Authors:** Yunmi Park, Jiyeon Shin, Ji Yi Lee

**Affiliations:** 1 Architectural and Urban Systems Engineering, College of Engineering, Ewha Womans University, Seoul 03760, Korea; ymp@ewha.ac.kr (Y.P.); jys.urban@ewhain.net (J.S.); 2 Department of Environmental Science and Engineering, College of Engineering, Ewha Womans University, Seoul 03760, Korea

**Keywords:** fine particulate matter, urban form, land cover, landscape ecology, Korea

## Abstract

Increasingly detrimental effects of fine particulate matter (PM) have been observed in Northeast Asia owing to its rapid economic development. Previous studies have found that dust, combustion, and chemical reactions are the major sources of PM; nevertheless, the spatial configuration of land use and land cover, which is of most interest to planners and landscape architects, also influences the PM levels. Here, we attempted to unveil the relationship between PM and different types of land use cover (i.e., developed, agricultural, woody, grass, and barren lands) in 122 municipalities of Korea. Landscape ecology metrics were applied to measure the spatial configuration of land use pattern and spatial lag models by taking into account the transboundary nature of air pollution, allowing us to conclude the following regarding PM levels: (1) the size of land cover type matters, but their spatial configuration also determines the variations in PM levels; (2) the contiguity and proximity of landcover patches are important; (3) the patterns of grasslands (e.g., simple, compact, and cluster (with large patches) patterns) and woodlands (e.g., complex, contiguous, and cluster (with large patches) patterns) considered desirable for minimizing PM are dissimilar in terms of contiguity.

## 1. Introduction

Concerns about particulate matter (PM)—in particular, PM_10_ and PM_2.5_ in this study—have been growing globally since it was found to have detrimental effects on well-being; PM can increase mortality as well as the incidence of cardiovascular and respiratory diseases [1]. Poor visibility and numerous hazes, associated with the presence of PM, often reduce agricultural productivity [2] and weaken the aviation and tourism industries [3]. Since the detrimental effects of PM were first unveiled through different types of research, local governments have been putting increasing efforts into improving the air quality. In this context, the extremely high PM concentrations in Northeast Asia are the cause of concern so that most countries in this region have established individual air quality standards for PM in ambient air and have actively run air quality monitoring and management activities for the control of PM (WHO, 2006). In Korea, for example, the Clean Air Conservation Act was wholly amended in 2007, and the Special Act on Metropolitan Air Quality Improvement was enacted in 2004 [4]. The series of legal enactments were a result of the increasing concerns about low air quality levels—which were the worst among the countries of the Organization for Economic Cooperation and Development (OECD) in 2018 [5]—which pose a health threat to people living in one of the world’s most densely populated countries. On top of some factors internal to each country, the rapid growth of industry and the resulting severe air pollution in Northeast Asia (e.g., Mongolia, China, and North Korea) have been drawing global attention.

Many previous studies have focused on the causes of PM growth. The results indicated a direct impact of suspension from the soil, resuspension from industrial dust and vehicles, and fossil fuel combustion [6]; moreover, meteorological conditions have been found to have a moderating effect [7,8]. The impacts of urban form and land cover on PM have been of particular interest to planners and landscape architects, given that land use plans and open-space planning seem to help in reducing or accelerating PM exposure. Previous studies have mostly provided information on the number of land cover patches in terms of percentage or acreage as well as on the number of certain types of patches. To fill existing knowledge gaps, we explored the influence of the spatial configuration of different land cover types on PM_10_ and PM_2.5_ atmospheric concentrations in 122 municipalities of Korea. The outcomes of this study are expected to serve as a useful guide for the integration of land use and atmospheric environment planning.

## 2. PM, Urban Form, and Landscape Ecology

“PM” refers to a mixture of solid pollutants (e.g., salts, organic compounds, dust, and metals) and liquid droplets. PM can be divided into PM_10_ or PM_2.5_ based on having a particle diameter [9] of ≤10 μm (“inhalable fine particles”) or ≤2.5 μm (“fine particles”), respectively. Since PM is small enough to penetrate the respiratory system, it has been attributed as the cause of chronic respiratory diseases, cardiovascular diseases, premature mortality, and adult diabetes, as well as some minor symptoms (e.g., cough, dyspnea, and wheezing) [10]. PM_10_ includes both natural (originating from the soil, dust, ash, metal oxides, pollen, or plant parts) and anthropogenic components; meanwhile, PM_2.5_ is dominated by sulfate, nitrate, ammonium, metal (e.g., Pb, Cd, V, Ni, Cu, and Zn), and NH^+^_4_ [11] compounds, which mainly originate from anthropogenic activities. Well-known, significant sources of PM_10_ are suspensions from soils disturbed by farming or mining as well as resuspensions of industrial dust from streets and roads by vehicles [10]. PM_2.5_, on the other hand, is mainly generated from the combustion of coal, oil, gasoline, transformation products of NO_x_ and SO_2_, or from smelters and steel mills [6]. PM concentrations are profoundly enhanced or mediated by meteorological conditions such as precipitation, high humidity, and wind speed [7,8]. The effect of temperature is still controversial, although few studies have shown its connection to seasonal and temporal variations in PM (e.g., positive in winter and negative in summer) [12,13].

The number of studies showing an association between urban form—often measured by “land cover and land use” (LCLU)—and PM has been growing. Since the major sources of PM are closely related to human activities (e.g., construction sites, roads, power plants, and automobiles), the association between developed areas and PM has received considerable scholarly attention. First, PM concentrations are likely to increase as cities increase in size [14]; second, population density [15] is positively correlated to PM levels; third, the higher the traffic volume and the lower the distance of the measurement location from roads, the higher the detected PM level [16]. Notably, urban sprawl is seen as a primary reason for traffic volume increase: developed areas tend to show higher PM concentrations [17]; in particular, an area including several energy-related industries, manufacturing industrial establishments, and combustion facilities have been identified as a primary source of PM [15,18]. The contribution of agricultural lands to PM has also been highlighted: increases in agricultural emission have been found to be closely related to growth in food production and population [19,20]; furthermore, it has been claimed that the burning of crop residues is a leading source of PM [21]. The role of vegetation in reducing air pollutants and in air purification has been examined for a long time. As Riondato, et al. [22] declared, vegetated areas can filter pollutants and effectively reduce PM concentrations. This is in accord with other studies which, in particular, examined leaf characteristics (e.g., cuticle chemical composition [23] and the wax layer [24]), tree canopy size, plant health condition [25], groove and trichome distribution [26], PM deposition velocity, the ground surface fraction of vegetation [27], and tree and shrub density [28]. Finally, barren deserts, in particular when subjected to high-speed wind capable of blowing dust and sand, can act as sources of PM [29]. Although these previous studies have adopted different sorts of statistical methods to observe and estimate the impact of certain types of LCLU on PM levels (e.g., longer-term trend monitoring, kriging, linear mixed-effects, or random forest), the land use regression (LUR) model remains the most popular for long-term predictions of PM concentrations. The LUR model considers land use attributes and other factors correlated to air pollution [30]; however, the distance from the emission source and the proportion of specific land uses have been examined the most.

Via the prism of landscape ecology, it has been possible to observe the spatial patterns of LCLU and their impact on PM since landscape ecology metrics quantitatively measure different sorts of landscape structures (i.e., patches under the same LCLU type) [31], and relevant software (e.g., FRAGSTATS) are readily available [32]. Thus, landscape ecology metrics have been frequently used to examine the association between land use patterns and local flooding [31], neighborhood satisfaction [33], streamflows [34], and walkability [35]. The association between land cover as measured by means of landscape ecology metrics and PM has been investigated, but often only by referring to urban green space [36,37], aggregated urban patches [38], and developed areas [39]. Moreover, in previous studies on this topic, most of the analyzed areas were located in China, followed by Iran, Germany, and Poland. As Bergin, et al. [40] stated, determining the association between land cover and PM at different geographic scales and in different contexts would broaden our understanding of the linkage between pollutants and human activities (which are reflected on LCLU). Thus, the identification of PM uniquely found in Korea could help generalize previous observations and determine the phenomena occurring locally due to specific air pollution characteristics. Considering the transboundary nature of air pollution, the application of spatial autoregressive models for LUR (which is a simple linear regression) or pairwise correlation in the majority of previous studies appears both theoretically and practically adequate.

## 3. Materials and Methods

**Figure 1:**
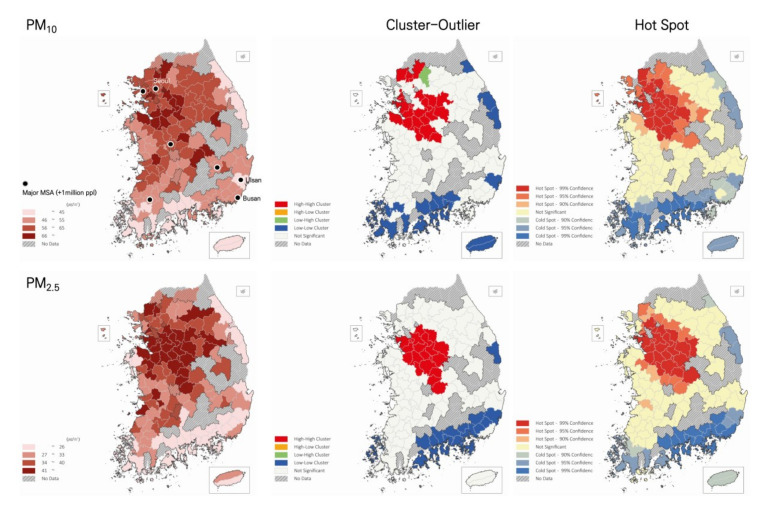
PM_10_ and PM_2.5_ distributions during the 2019 peak season.

The study area covered 122 out of 165 municipalities in Korea (Figure 1), which formed the dataset for our analyses. In this study, we mainly explored the average PM_10_ and PM_2.5_ levels over four months from December 2018 to March 2019. These months were selected because PM concentrations are highest during the winter season, and the results of targeted analyses would allow the placement of strong management policies by the government.

Land cover data with 30 m resolution were retrieved in 2019 by the Ministry of Environment to identify developed lands, agricultural lands, woodlands, grassland, and barren lands. To identify the spatial configuration of each land cover type, landscape ecology metrics measures to represent the area (total area), shape (large patch index), size of the largest patch (largest patch index), proximity (mean proximity), connectivity (mean contiguity), and diversity of each type of land cover (Shannon’s diversity index) were calculated using ArcMap 10.4.1 and FRAGSTATS v4.2.1 (see Table 1 for the detailed measurements of each metric). Data on possible correlated factors (relative to the year 2019) were collected based on the previous literature. These included data on population [19], manufacturing employees and companies [15], the total number of cars [41], road length [42], the number of businesses generating >100,000 tons of pollutants per year [18], and meteorological data (i.e., temperature, humidity, rainfall, temperature, and wind speed) [12]. Wind direction data were excluded since the values retrieved from monitoring stations located in different parts of the same municipality differed considerably. Instead, the longitude and latitude of each municipality were taken into account to investigate the influence of polluted air from adjacent countries such as China or North Korea [43]. The data were retrieved from Statics Korea, the Traffic Accident Analysis System, the Korea Meteorological Administration, and the 2019 Air Emission Source Survey. Spatial lag models (SLM) were used for the analyses (for the choice of SLM, please see Appendix A).

The patch densities, shapes, and fractal dimensions, as well as the proportion of each land cover, were excluded from the analyses due to multicollinearity. A correlation was considered “high” for r > 0.8 (*p* < 0.05). The standard values were set slightly high since the landscape ecology metrics measured slightly different aspects of patches so that the correlation coefficients typically showed high values. We did not create a composite index based on these variables because it would not have provided any significant meaning, and the configuration of land cover was the major point of interest in this study. Meanwhile, the composite score of urbanization achieved by factor analysis that includes population, the total number of cars, employers, companies, and the total length of roads were used to avoid simultaneous consideration of many individual variables. The latitude and longitude of municipalities, emission sources, urbanization, wind speed, and temperature–precipitation (i.e., the composite score of temperature and precipitation obtained by factor analysis) were included in the final models. For better comparison, the variables were maintained constant across different land cover models.

## 4. Results

### 4.1. Descriptive Statistics

As shown in Figure 1, high concentrations of PM_10_ and PM_2.5_ were detected in inland areas, while coastal cities in the east and south showed lower PM levels. In particular, high–high clusters—a statistically significant cluster of relatively high values of PM levels compared to surrounding areas—and hot spots—identifying a group with high value within the study area—were identified in the Chungcheong and Southern Gyeonggi provinces, which include 314 industrial parks as of 2020 and are closely located in Seoul metropolitan area. The Chungcheong province has a basin shape since it is surrounded by mountain ranges. Most of the South and East Coast areas showed low concentrations of PM_10_ and PM_2.5_; in particular, low–low clusters and cold spots were observed in the South Coast area, which includes Gyeonsangnam, Jeollanam, Busan, and Ulsan, for a total of 227 industrial parks. Major metropolitan areas, including Seoul, which were expected to show high concentrations of PM, were not a part of high–high clusters. Clusters of PM_10_ and PM_2.5_ showed similar patterns, but the shape of the PM_2.5_ high–high cluster seemed narrower, while the PM_10_ high–high cluster was found to stretch toward the west shore and included four municipalities located at the border with North Korea. An outlier, low–high cluster—an area with relatively low PM levels compared to surrounded cities—that was observed in Gapyeong-gun showed relatively low PM_10_ concentrations when compared with the surrounding areas. These low concentrations might be due to the uniqueness of the area, which is characterized by a high proportion of woodland, a low number of emission sources (i.e., only one industrial complex), and is surrounded by high mountains. By contrast, other border areas (e.g., Paju and Yangju) showed high PM_10_ concentrations due to their higher number of industrial parks and to the ongoing construction of a new town.

As shown in Table 2, the total woodland area represented most of the land cover in S. Korea, followed by agricultural land, developed land, grassland, and barren lands. Notably, woodland covered an area ten times larger than that of developed land. On average, the edge density (ED) of agricultural land was the highest (56.87), while that of barren land was the lowest (13.33); agricultural land was characterized mainly by very fine and scattered spatial patterns. The large patch index (LPI) that refers to the proportion of the single largest patch in the area suggested that the largest patches were represented by woodland (comprising on average 33.62% of each studied municipality). The mean contiguity and the proximity ranged between 0.05–0.29 and 0.76–333,784.80, respectively. Woodland showed the highest mean contiguity and proximity, which implied that woodland was more likely to be connected, complex, and clustered by large patches. The average value of the Shannon diversity index (SHDI) was 0.53, implying the presence of diverse land cover types; populous cities tended to have more diverse land covers (r = 0.26, *p* = 0.00). Notably, one municipality had about 31 emission sources, which generated >100,000 tons of pollutants per year on average. Overall, the mean wind speed, temperature, and precipitation over the four winter months were 1.93 m/s, 2.03 °C, and 25.47 mm, respectively.

### 4.2. SLM Results

The separate land cover model illustrated that LCLU and its spatial patterns impacted the PM_10_ and PM_2.5_ levels (Table 3). As expected, the spatial autoregressive parameter (*Rho*) was highly significant across the models, and the explanatory power of each model was 69–73% and 70–73%, respectively. The adjusted R^2^ values of PM_10_ and PM_2.5_ SLM obtained by considering only the control variables (i.e., base model) were 65% and 68%, respectively. This implies that other than the land cover, the control variables (i.e., longitude, latitude, urbanization, emission sources, wind speed, temperature, and precipitation) were the major driving factors of the observed PM levels. The results of the PM_10_ base model indicated that longitude (coef. = −0.02, *p* = 0.09), emission sources (coef. = 1.73, *p* = 0.03), and wind speed (coef. = −6.00, *p* = 0.00) were all statistically significant factors at the 0.5 significance level. Meanwhile, the results of the PM_2.5_ base model indicated longitude (coef. = −0.01, *p* = 0.07), emission sources (coef. = 1.27, *p* = 0.06), wind speed (coef. = −4.13, *p* = 0.01), and temperature (coef. = −0.98, *p* = 0.09) were significant factors.

**Figure 2:**
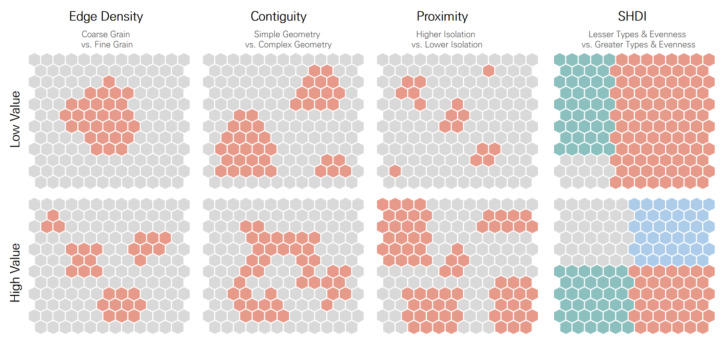
An intuitive interpretation of landscape ecology metrics (colored pattern refers to different land cover types of interest).

The results of the SLM for each land cover type were interpreted at the 0.1 significance level and intuitively read, as shown in Figure 2. The mean contiguity value increased in the case of contiguous patches and complex shapes. A higher value of proximity indicated that the patch was surrounded by many larger patches, which would be close to each other (i.e., low isolation). In the case of a single, large patch, the mean proximity value would be lower. Notably, higher Shannon’s index values identified a higher number of patch types and evenness among those patch types at the same time. The effects of different land covers on PM_10_ and PM_2.5_ were quite distinct. Overall, emission sources, wind speed, and temperature showed significant associations with the PM levels. Latitude was negatively associated (or approached significance) in the PM_2.5_ model. In the developed area model, contiguity (coef._pm10_ = 62.31, coef._pm2.5_ = 32.08) and SHDI (coef._pm10_ = 9.47, coef._pm2.5_ = 4.62) showed positive influences on the PM_10_ and PM_2.5_ levels. The larger the agricultural land, the lower was the PM_10_ and PM_2.5_ levels (coef._pm10_ = −0.22, coef._pm2.5_ = −0.18). The ED was also found to contribute positively to the PM_10_ levels (coef._pm10_ = 0.12) and approached significance in the PM_2.5_ model. By contrast, the high values of contiguity (coef._pm10_ = −81.81, coef._pm2.5_ = −36.77) and proximity (coef._pm10_ = −2.12, coef._pm2.5_ = −1.16) associated with woodlands contributed to the decrease in PM_10_ and PM_2.5_ levels. In the grassland model, an increase in the mean contiguity would lead to significant growth of both types of PM (coef._pm10_ = 61.65, coef._pm2.5_ = 92.01), while an increase in the mean proximity would lead to a reduction in both types of PM (coef._pm10_ = −2.92, coef._pm2.5_ = −2.75). SHDI was found to be a significant factor and have a positive effect on PM only in developed and barren lands.

## 5. Discussion

Interest in the mediation of PM levels has been growing due to the detrimental effects on public health and the environment observed [36]. Apart from environmental scientists, scholars and practitioners in the fields of planning, design, and landscape architecture have also attempted to examine how the built environment could mediate PM levels, e.g., through the modification of street morphology, the control of building height, vehicle traffic, and coal-mining power plants, and the adaptation to green infrastructure [44]. The results of this study can also be of use to planners and landscape architects interested in the importance of the spatial configuration of urban form and can guide the formulation of air pollution management-related enforcements and principles.

The results of the SLM indicate that the PM influences neighboring municipalities: the diffusion effect of PM between adjoining neighbors was empirically tested. However, the interpretation of the term “adjoining” should be cautiously interpreted. In this study, the distance band of 100 km was applied to detect the spatial autocorrelation because the first peak distance of the spatial autocorrelation was observed around this distance for both PM. When considering that the average (east-west) width and (south-north) length of Korea are 300 and 1000 km, respectively, it can be assumed that an increase in PM of a particular city within the country would directly lead to a similar increase in many of its surrounding cities. The analysis of hot spots and cluster outliers also indicated that the levels of PM were highly clustered and exchange influences in general. As already mentioned by Bergin, West, Keating and Russell [40] and others (Bae et al., 2020; Kim et al., 2017), these results imply that PM can be transported across regions. This finding could also be explained by the secondary formation of PM within the same air mass areas (Choi et al., 2021). Future study is encouraged to consider these two main driving factors of PM (long-range transport and/or secondary formation of PM) in the SLM model. In particular, future studies could investigate how results can vary by adopting different spatial weight matrices; in other words, using different distance bands.

Our results indicated that the size of some types of land cover mattered, but not always, and that this was less important than contiguity and proximity. The total area is well known for being one of the most important and useful pieces of information for determining the dominance of certain land cover types; however, only the amount of agricultural land seemed to influence the PM_10_ and PM_2.5_ levels. Developed lands with high contiguity generally showed higher PM levels, as opposed to woodlands: when woodland areas had complex, contiguous, and less-isolated shapes, they were associated with lower PM levels. This is possibly due to a relatively high land roughness related to the contiguous edges of the forest, such as the fortress form, which would help capture airborne pollutants more efficiently [45]. It is also well known that a high density of leaves and canopy could block and rub the particles and that the sedimentation process would be accelerated in forests [28]. On the contrary, different types of urban structures with different sizes, heights, and shapes in developed areas—where land cover data cannot be directly measured through satellite images—may decrease the ventilation ability [39] and act as wind barriers [36]. To combine previous studies and current findings, creating multiple rows of green buffers (by planting street trees) would mediate the PM levels in such instances.

Large and coarse patches of agricultural land (e.g., rice paddies, fields, greenhouses, and orchards) are needed to decrease current PM. A decrease in the number of activities, urban structures, or emission sources in rural communities would cause a considerable lowering of the PM and allow a much freer airflow compared to what occurs in highly developed areas [46]; also, crops and fruit trees may absorb some PM. However, this does not necessarily infer that creating a large and coarse agricultural land by clearcutting forests reduces the PM level. The results should be interpreted carefully in relation to other areas of use. This could be a specific phenomenon that occurred in Korea since agriculture is not the main industry of S. Korea, and industrial farming is rarely observed. Rather, the products of farming activities (e.g., soil dust, soot, and grain dust from soil preparation, harvest operations, or crop residue burning) are less well managed by small-scale farmers who possess less appropriate machinery and maintain a process that is associated with air pollution. In particular, crop residue burning and agricultural byproducts have been claimed to be among the major sources of reparable PM [47]. For this reason, the Korean government has carried out special crackdowns on illegal incineration in rural areas during the winter and early spring seasons. Probably other countries such as India, Mongolia, or China would take some lessons from this action.

When grassland patches have simple and compact shapes and are clustered with large patches, PM tends to be relatively low. No previous studies have observed the spatial patterns of grasslands and their impact on PM, but it can be assumed that grassland has to be over a certain size to be effective: grasslands have a lower capacity to capture a large number of particles compared to dense forests with broad-leaved trees [48]. Sediment, however, would encounter fewer obstacles in an open lawn than in a forest so that wind would easily transport the particles to a new location [49]. This suggests that having a single large open green space would be beneficial. Future studies may describe, in detail, the effect of different types of vegetation (e.g., trees, grassland, parks, and open space). However, this distinction needs to be determined carefully and be subsequently supported by empirical research.

As expected, weather conditions and location can all play critical roles in determining the PM levels. In particular, a place characterized by relatively slow winds and low temperatures is likely to show higher PM concentrations due to a low dispersion of particles and heat. Different from our hypothesis, urbanization did not show any statistically significant impact on PM level. Apparently, the number of old and heavy-duty cars would be more influential than the total number of cars. Urbanized areas would have more manufacturing jobs and companies, but most have fewer air-polluting industries than compared with rural communities. Alternatively, this might be explained by the fact that sufficient monitoring and management systems may exist in large urbanized cities. In fact, in Seoul (i.e., the largest city in Korea), the PM values slightly exceeded the country’s mean, and the other top five largest cities of the country had below-average PM levels. Emission sources are both positive and significant factors, and densely developed cities have relatively fewer major emission sources. Notably, the Seoul Metropolitan Area Readjustment Planning Act controlled the total built area for remodeling and construction of factories within the Seoul metropolitan area (inhabited by 20 million people), and the total allowed amount of land is renewed every three years. That is why hot spots of PM were detected in the central inland area—that is relatively less urbanized—close to the Seoul metropolitan area with more flexibility to build new factories. Meanwhile, the high–high cluster of PM_10_ and the relatively high concentration of industrial parks near the west border with North Korea reflects the efforts of the government to prepare for a future unification of South and North Korea. Industrial parks between Seoul (S. Korea) and Kaesong (N. Korea) were planned and built in the 1990s and 2000s jointly with the Special Economic Zone of North Korea and have been run by Korea. The construction of the city of Paju new town, completed in 2009, contributed to the transformation of historically agricultural land into developed land. The possible transport of PM from North Korea should also not be ignored; considering the proximity of North Korea to the northern municipalities of Korea and the large emission of air pollutants by unit energy consumption (mainly of biomass and coal) in North Korea, air pollutants emitted from the latter country might influence the air quality in neighboring areas [43]. Future studies may hence investigate the impacts of North Korea and LCLU on PM levels in Korea.

## 6. Conclusions

This study illustrates the importance of the spatial configuration of different land-use types in mediating or accelerating the increase in PM levels. In particular, it is recommended that planners and landscape architects take these findings into account when preparing land use plans and green space plans. A mere enlargement of vegetated areas or agricultural lands cannot be considered a remedy for air pollution in cities; rather, existing small patches of agricultural land should be carefully monitored. Moreover, the design of woodland and grassland areas should be carefully implemented, and strip development should be hindered. Moreover, the balance between different land-use types should be carefully considered. Despite meaningful lessons for planners and landscape architects or related fields, there are several tasks that future studies can discuss further. We recommend that future studies take a microscopic perspective and not remain at city-level analysis as was the case with our study. As found in the previous literature, the proximity of PM emission sources, the characteristics of land cover in terms of development (e.g., high, medium, or low), and microclimate are important features to consider for determining PM levels. While 30 m medium-resolution land cover data could capture the total ground and dominant features—however, they could miss details at a level that is not fine enough to distinguish individual objects, or that misses small patches surrounded by the dominant features: for example, street trees in a predominantly developed area. Therefore, future studies could use high-resolution imageries and locational datasets to take a microscopic perspective and should not remain at the city-level analysis as in our study. Even though the winter peak months were taken as an observation window, corresponding to when the PM concentration has been the worst in S. Korea, different time frames would be applicable to other countries based on their own geographical, meteorological, and contextual conditions.

## Figures and Tables

**Table 1:** Variables and Measurements.

Variable	Measurement
Total Area	The total land area of certain patch types within the studied unit (10,000 m^2^)
Edge Density (ED)	$\mathrm{ED}=\frac{E}{A}$ E = sum of edges in the landscape (A)
Largest Patch Index	% of landscape comprised in the single largest patch
Mean Contiguity	Mean value of the contiguity index of patches within the landscape: $\mathrm{Contig}=\left(\;\frac{\left[(\sum_{r=1}^{z}c_{ijr})/a_{ij}\right]-1}{\text{v}-1}\right)$ c_ijr_ = contiguity value for pixel r in patch ij, a_ij_ = area of patch ij in terms of cell number, v = sum of values in a 3-by-3 cell template
Mean Proximity	$\mathrm{PX}=\sum_{i=1}^{n}\frac{S_{i}}{z_{i}}$ S_i_ = area, z_i_ = edge to edge distance from patch i to its nearest neighbor patch within the buffer
Shannon’s Diversity Index (SHDI)	$H^{\prime }=-\sum_{i=1}^{c}p_{i}\ln(p_{i})$ c = number of classes in the landscape, p_i_ = proportion of area in class i
Urbanization	Urbanization composite score calculated by considering the population, manufacturing employees and companies, cars, and road length
Emission Source	Total number of businesses generating >100,000 tons of pollutants per year
Wind Speed	Average wind speed during the peak season (m/s)
Temperature	Average temperature (December–March) (°C)
Precipitation	Average precipitation (December–March) (mm)

**Table 2:** Descriptive Statistics.

Variables		Mean	SD	Min	Max
Total Area	Developed	3987.27	4902.77	66.15	35,116.47
	Agricultural	12,939.91	9182.47	69.21	45,156.42
	Woody	40,687.93	30,598.34	438.39	162,833.00
	Grass	2020.31	2287.46	72.18	17,133.48
	Barren	1077.41	974.26	17.19	8054.91
Edge Density	Developed	23.93	17.05	1.71	77.22
	Agricultural	56.87	21.74	5.67	98.29
	Woody	46.15	12.2	20.04	80.68
	Grass	27.59	17.02	2.03	100.88
	Barren	13.33	10.48	2.55	71.92
Largest Patch Index	Developed	4.21	9.24	0.05	55.52
	Agricultural	5.89	8.61	0.08	51.28
	Woody	33.62	24.7	0.93	91.82
	Grass	0.26	0.55	0.00	4.66
	Barren	0.22	0.31	0.01	1.67
Mean Contiguity	Developed	0.16	0.03	0.10	0.24
	Agricultural	0.2	0.01	0.15	0.23
	Woody	0.21	0.03	0.16	0.29
	Grass	0.12	0.03	0.05	0.23
	Barren	0.15	0.02	0.08	0.21
Mean Proximity	Developed	1129.84	6802.14	4.06	73,983.01
	Agricultural	2922.12	6905.97	8.76	50,311.91
	Woody	38,609.95	64,603.39	73.2	333,784.80
	Grass	10.05	44.77	0.76	481.01
	Barren	6.48	6.75	0.95	41.07
Shannon’s Diversity Index		0.53	0.16	0.15	0.87
Urbanization		−0.00	1.00	−0.55	7.97
Emission Source		30.89	42.27	0.00	210.00
Wind Speed		1.93	0.79	0.55	4.60
Temperature		2.03	2.10	−2.55	6.80
Precipitation		25.47	9.29	12.81	75.88

**Table 3:** Results of the SLM.

Variables		Developed Land		Agricultural Land		Woodland		Grassland		Barren Land	
		PM_10_	PM_2.5_	PM_10_	PM_2.5_	PM_10_	PM_2.5_	PM_10_	PM_2.5_	PM_10_	PM_2.5_
Total Area	Coef.	589.70 ^†^	137.34 ^†^	−0.22 **	−0.18 **	−0.00	0.00	45.60 ^†^	−174.88 ^†^	−61.26 ^†^	−291.94 ^†^
	Std.Err.	0.99	0.82	0.00	0.00	0.00	0.00	0.80	0.65	1.02	0.83
ED	Coef.	−0.00	0.03	0.12 **	0.06	0.01	−0.00	1.68 ^†^	0.46 ^†^	0.61 ^†^	−0.15 ^†^
	Std.Err.	0.06	0.05	0.05	0.04	0.04	0.04	1.51	1.20	1.40	1.14
Largest Patch Index	Coef.	-	-	-	-	-	-	0.70 ^†^	0.52 ^†^	0.70 ^†^	0.54 ^†^
	Std.Err.	-	-	-	-	-	-	0.80	0.63	0.84	0.68
Mean Contiguity	Coef.	62.31 **	32.08 *	−8.62	−7.64	−81.81 **	−36.77 **	61.65 *	92.01 **	48.21	5.34
	Std.Err.	23.92	19.57	34.37	28.27	22.36	19.09	35.06	27.93	29.45	23.95
Mean Proximity ^†^	Coef.	−0.16	−0.61	0.71	0.72	−2.12 **	−1.16 **	−2.92 **	−2.75 **	−1.65	−1.33
	Std.Err.	0.55	0.45	0.56	0.46	0.64	0.55	1.41	1.13	1.35	1.09
SHDI	Coef.	9.47 **	4.62	3.53	1.08	−1.06	−0.70	6.00	1.21	8.72 **	5.67 *
	Std.Err.	3.57	2.88	3.94	3.21	4.17	3.56	3.66	2.90	4.05	3.26
Longitude	Coef.	−0.00	0.00	0.01	0.00	−0.00	0.00	0.00	−0.00	0.00	0.00
	Std.Err.	0.00	0.00	0.00	0.00	0.00	0.00	0.00	0.00	0.00	0.00
Latitude	Coef.	0.00	−0.01	−0.00	−0.01 *	−0.00	−0.01 *	−0.00	−0.02 **	−0.00	−0.01
	Std.Err.	0.00	0.00	0.00	0.00	0.00	0.00	0.00	0.00	0.00	0.00
Urbanization ^†^	Coef.	−3.08	−0.56	1.66	0.93	−0.76	−0.78	−1.18	−1.02	−2.20	−1.00
	Std.Err.	1.91	1.59	1.86	1.53	1.39	1.20	1.44	1.16	1.48	1.22
Emission	Coef.	1.31 **	1.08 **	0.72	0.67	1.62 **	1.21 **	1.46 **	1.35 **	1.56 **	1.18 **
	Std.Err.	0.60	0.50	0.62	0.51	0.55	0.47	0.62	0.49	0.64	0.52
Wind Speed ^†^	Coef.	−6.62 **	−4.40 **	−5.81 **	−3.88 **	−6.12 **	−4.16 **	−5.11 **	−3.06 **	−5.53 **	−3.25 **
	Std.Err.	1.63	1.31	1.59	1.29	1.54	1.31	1.61	1.27	1.65	1.33
Temperature ^†^	Coef.	−1.23 **	−1.27 **	−1.37 **	−1.36 **	−1.28 **	−1.33 **	−1.78 **	−1.69 **	−1.40 **	−1.40 **
	Std.Err.	0.52	0.42	0.52	0.42	0.52	0.43	0.54	0.42	0.55	0.43
Precipitation ^†^	Coef.	−2.97	0.96	−2.04	0.86	−1.34	1.47	−0.25	1.82	−1.08	2.18
	Std.Err.	2.59	2.05	2.48	1.99	2.35	1.95	2.51	1.95	2.58	2.05
Constant	Coef.	19.39	0.25	31.93	8.08	77.05	29.29	27.26	5.88	20.60	2.77
	Std.Err.	16.24	11.16	17.88	12.30	19.07	14.06	16.01	10.60	16.13	10.99
*Rho*		0.42 **	0.74 **	0.42 **	0.70 **	0.40 **	0.71 **	0.33 **	0.62 **	0.46 **	0.74 **
N		122	122	122	122	122	122	122	122	122	122
Adjusted *R*^2^		0.70	0.70	0.70	0.70	0.73	0.71	0.69	0.73	0.69	0.70

^†^ refers to a log-transformed variable; ** refers to *p* < 0.05 and * to *p* < 0.1.

## Data Availability

Not applicable.

## Appendix A. Model Choice

The ordinary least squares (OLS) method and the geographically weighted regression (GWR) were initially applied; GWR was used to test the presence of possible spatial nonstationary effects across the study areas. Subsequent significance tests for the nonstationarity of the parameter estimates (*p* < 0.05) favored the results of the OLS method over those of the GWR. After applying the OLS method, spatial autocorrelations were verified between the residuals. The Lagrange multiplier (LM) test suggested the use of spatial lag models (SLM) over spatial error models. Moreover, the distance band of the spatial weight matrix was set as 100 km since the incremental spatial autocorrelation indicates that clustering peaked around 100 km in both PM_10_ and PM_2.5_. Afterward, the SLM models were applied separately for different land cover types.
